# Combined Brown syndrome and superior oblique palsy without a trochlear nerve: case report

**DOI:** 10.1186/s12886-017-0553-9

**Published:** 2017-08-25

**Authors:** Hee Kyung Yang, Jae Hyoung Kim, Ji-Soo Kim, Jeong-Min Hwang

**Affiliations:** 1 Department of Ophthalmology, Seoul National University College of Medicine, Seoul National University Bundang Hospital, 166, Gumiro, Bundang-gu, Seongnam, Gyeonggi-do 463-707 South Korea; 2 Department of Radiology, Seoul National University College of Medicine, Seoul National University Bundang Hospital, Seongnam, South Korea; 3 Department of Neurology, Seoul National University College of Medicine, Seoul National University Bundang Hospital, Seongnam, South Korea

**Keywords:** Brown syndrome, Superior oblique palsy, Trochlear nerve

## Abstract

**Background:**

Congenital Brown syndrome is characterized by limited elevation particularly during adduction. The pathogenesis of congenital Brown syndrome is still controversial.

**Case presentation:**

A 6-year-old boy had been tilting his head to the left since infancy. He showed right hypertropia (RHT) of 2 prism diopters (Δ) in the primary position. He showed RHT 6Δ in right gaze, RHT 2Δ in left gaze, RHT 12Δ in right head tilt, and orthotropia in left head tilt. The right eye showed limitation of elevation and depression on adduction, and the left eye showed overdepression on adduction. MR images showed an absent right trochlear nerve with a hypoplastic ipsilateral superior oblique muscle.

**Conclusions:**

Congenital Brown syndrome may be associated with an absent trochlear nerve and hypoplastic superior oblique muscle suggesting an etiologic mechanism of congenital cranial dysinnervation disorder.

## Background

Congenital Brown syndrome is characterized by limited elevation particularly during adduction from mechanical causes [1]. The pathogenesis of congenital Brown syndrome is still controversial, and we have previously found normal-sized trochlear nerves and superior oblique (SO) muscles on high-resolution magnetic resonance imaging (MRI) in nine patients with congenital Brown syndrome [2]. In contrast, Kaeser et al. reported an absent trochlear nerve with normal sized SO muscles [3], and Ellis et al., a hypoplastic SO in congenital Brown syndrome without confirming the status of the trochlear nerve, suggesting the etiology as a variant of congenital cranial dysinnervation disorders (CCDD) [4]. Recently we found a patient with limited elevation and depression during adduction suggesting congenital Brown syndrome with concurrent SO palsy in the same eye, who had no visible trochlear nerve on the ipsilateral side together with a hypoplastic SO confirmed by high-resolution MRI.

## Case presentation

**Fig. 1:**
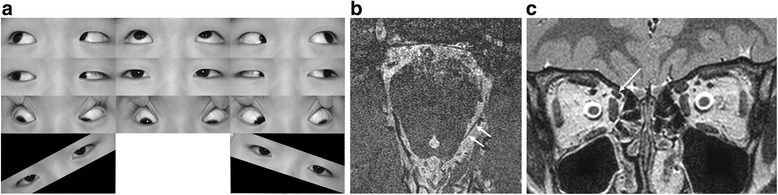
**a** Ocular versions demonstrating limitation of elevation and depression on adduction and downgaze of the right eye, and overdepression on adduction of the left eye. During adduction and elevation, widening of the lid fissure is clearly distinct in the right eye compared to the left eye. **b** The left trochlear nerve (arrows) is well identified, but the right is not observed. **c** The right superior oblique muscle is hypoplastic (arrow) compared to the left

A 6-year-old had been tilting his head to the left since infancy. His past medical history was unremarkable. Best-corrected visual acuities were 20/30 in both eyes. He showed right hypertropia (RHT) of 2 prism diopters (Δ) on alternate prism and cover test in the primary position at distance and at near. He showed RHT 6Δ in right gaze, RHT 2Δ in left gaze, RHT 12Δ in right head tilt, and orthotropia in left head tilt. The right eye showed limited elevation (−3) and depression (−4) on adduction, and the left eye showed overdepression (+3) on adduction. The right eye also showed mild limitation of elevation (−1) on abduction causing an overelevation (+1) on adduction in the contralateral left eye. Widening of the lid fissure was found during adduction and elevation in the right eye, and this was not found in the left eye (Fig. 1a). There was no subjective torsion measured with the double Maddox rod test. Fundus photographs with an internal fixator featured 3 degrees of extorsion in the right eye and no torsional abnormality in the left eye. Forced duction test revealed a mild restriction during adduction and elevation in the right eye. Saccadic velocities measured with infrared video-oculography were within normal ranges and symmetric in both eyes in the horizontal, vertical and diagonal directions.

T2-weighted images were obtained with 0.25-mm thickness for the trochlear nerve, and 1.4-mm thickness for the oculomotor nerve and abducens nerve in the basal cistern using a 3-Tesla MRI system (Intera Achieva; Philips Healthcare, Best, the Netherlands). The right trochlear nerve was absent, and ipsilateral SO muscle was hypoplastic (Figs. 1b, c). The oculomotor and abducens nerves were of normal size on both sides. All the other extraocular muscles except the SO were normal in size and symmetric on both sides. The distance between the annulus of Zinn and the trochlea was 31 mm in both eyes.

## Discussion

In this report, we clearly showed that the right trochlear nerve was absent and ipsilateral SO muscle was hypoplastic, thus supporting that CCDD is one of the etiologic mechanisms of Brown syndrome.

Kaeser et al. [3] first reported the association of congenital Brown syndrome and an absent trochlear nerve. They reported bilaterally absent trochlear nerves in two patients and a unilaterally absent trochlear nerve in two other patients [3]. Interestingly, the SO was not hypoplastic in any of their four patients suggesting the possibility of an alternative innervation [3]. However, they used a 1.5-Tesla magnetic resonance unit (Siemens, Erlangen, Germany) [3], and in our experience, the trochlear nerve was visible only by using a voxel smaller than the diameter of the trochlear nerve in a high-resolution 3-Tesla system [5]. In our previous studies on congenital superior oblique palsy, all the patients without a trochlear nerve were unilaterally affected [5–9], and their paretic SO areas and volumes were significantly smaller than the normal side [9]. In contrast, in patients with a normal trochlear nerve, the paretic SO areas and volume showed no significant difference with the normal side [9]. Therefore, it is unlikely to observe a normal-sized SO in any patient without a trochlear nerve, particularly if the SO palsy is congenital. The possibility of synkinesis may arise in such conditions with a normal-sized SO and absent trochlear nerve, [3] however, the size of the SO may still be smaller than the contralateral normal side as found in our case.

The pathologic findings that have been found in Brown syndrome include enlarged and irregular tendon-trochlea complex [10], hypoplasia of the paretic SO [4, 11], restrictive fibrous adhesions to the posterior globe [12], increase of the distance between the annulus of Zinn and the trochlea [13], and bifid scleral insertion of SO [14]. Our patient showed hypoplasia of the paretic SO and an absent trochlear nerve, however, he also showed minimal extorsion in the paretic eye, and widening of the lid fissure during ipsilateral adduction and elevation, which are all indirect signs of an anomalous innervation of the SO muscle by fibers of the oculomotor nerve originally innervating the inferior oblique (IO) muscle [15]. Simultaneous contraction of the SO with IO muscles might produce limitation of elevation in adduction and minimal extorsion, or even intorsion [15]. The more anomalous branches of the IO supplying oculomotor nerve misdirected to the SO, the more intorsion might be induced. However, to date, direct evidence of synkinetic innervation of the SO muscle by branches of the oculomotor nerve has not been obtained histologically or with electromyography [15].

Brown syndrome could be classified as mild (no hypotropia in primary or adducted position), moderate (hypotropia in adducted position), and severe (hypotropia in primary position) [16]. Jampolsky classified Brown syndrome as true Brown syndrome (no hypotropia in primary position or down gaze) and Brown syndrome plus (vertical deviation in primary position or adduction with/without head posture) [17]. With written and oral communications, Stager et al. [18] termed Brown syndrome plus as Brown syndrome worse than mild Brown syndrome, which showed no vertical deviation in any of the horizontal gaze positions. Our patient corresponded to mild or true Brown syndrome because he showed a right hypertropia of 2 Δ in the primary position. Forced duction test revealed a mild restriction in the field of action of the right SO. In addition, he also showed a mild limitation of elevation during abduction, which also could be helpful to rule out the possibility of a right IO palsy. An anomalous innervation of the SO muscle by fibers of the oculomotor nerve originally innervating the IO muscle may simulate Brown syndrome showing a positive forced duction test.

Our patient showed not only Brown syndrome, but also superior oblique palsy mimicking canine tooth syndrome or dog-bite syndrome. Canine tooth syndrome is an ocular motility disorder characterized by limited elevation and depression on adduction [19]. Canine tooth syndrome is originally reported with a dog bite of the superior oblique trochlea region, but subsequently with head injury [20], superior oblique myocysticercosis [21], iatrogenic injury to the superior oblique tendon such as sinus surgery [22] or hook injury [23]. Our patient did not have any history of injury or infection, and proved the possibility of trochlear nerve agenesis as the etiology of canine tooth syndrome.

CCDD represent a group of neurodevelopmental diseases of the brainstem and cranial nerves [4]. As Ellis et al. assumed from their three patients with SO hypoplasia [4], one of the etiologic mechanisms of Brown syndrome may include CCDDs caused by an absent trochlear nerve. However, the underlying etiologies remain unknown in most of the cases and are yet to be elucidated. The positive forced duction test and spontaneous resolution found in some cases suggest that innervational and structural mechanisms may both be responsible for this phenomenon [16].

## Conclusion

Congenital Brown syndrome may be associated with an absent trochlear nerve and SO hypoplasia suggesting an etiologic mechanism of CCDD.

## Abbreviations

CCDD: congenital cranial dysinnervation disorder
MRI: magnetic resonance imaging
RHT: right hypertropia
SO: superior oblique
